# Specificity and heregulin regulation of Ebp1 (ErbB3 binding protein 1) mediated repression of androgen receptor signalling

**DOI:** 10.1038/sj.bjc.6602257

**Published:** 2004-12-07

**Authors:** Y Zhang, A W Hamburger

**Affiliations:** 1 Greenebaum Cancer Center, University of Maryland School of Medicine, Baltimore, MD, USA; 2 Department of Pathology, University of Maryland School of Medicine, Baltimore, MD, USA

**Keywords:** prostate cancer, androgen receptor, ErbB receptors, Ebp1

## Abstract

Although ErbB receptors have been implicated in the progression of prostate cancer, little is known about proteins that may mediate their interactions with the androgen receptor (AR). Ebp1, a protein cloned via its association with the ErbB3 receptor, binds the AR and inhibits androgen-regulated transactivation of wild-type AR in COS cells. As the complement of coregulators in different cells are important for AR activity, we determined the effect of Ebp1 on AR function in prostate cancer cell lines. In addition, we examined the regulation of Ebp1 function by the ErbB3/4 ligand heregulin (HRG). In this study, we demonstrate, using several natural AR-regulated promoters, that Ebp1 repressed transcriptional activation of wild-type AR in prostate cancer cell lines. Downregulation of Ebp1 expression in LNCaP cells using siRNA resulted in activation of AR in the absence of androgen. Ebp1 associated with ErbB3 in LNCaP cells in the absence of HRG, but HRG induced the dissociation of Ebp1 from ErbB3. In contrast, HRG treatment enhanced both the association of Ebp1 with AR and also the ability of Ebp1 to repress AR transactivation. These studies suggest that Ebp1 is an AR corepressor whose biological activity can be regulated by the ErbB3 ligand, HRG.

Prostate cancer is the second most prevalent cancer among men in the United States and ranks second to lung cancer in terms of annual mortality (Weir *et al*, 2003). Prostate cancer begins as an androgen-dependent tumour that undergoes clinical regression in response to pharmacological and surgical strategies that reduce testosterone concentration. Despite this treatment, the cancer eventually regrows as an androgen- or hormone-independent tumour (Feldman and Feldman, 2001). Microarray analysis of both androgen dependent and independent tumour xenografts (Amler *et al*, 2000; Mousses *et al*, 2001) and human prostate cancer samples (LaTulippe *et al*, 2002) during disease progression has identified several candidate targets, including the AR itself, for prostate cancer therapy and diagnosis associated with the androgen independent phenotype. Thus, aberrant changes in AR signalling are likely to play a role in the progression to androgen independence (Grossmann *et al*, 2001). Chen *et al* (2004) showed, using microarray-based profiling of isogenic prostate cancer xenografts, that increases in AR mRNA were the only changes consistently associated with development of resistance to antiandrogen therapy, providing a strong rationale for targeting the downregulation of androgen receptor (AR) activity in the treatment of advanced prostate cancer.

The potential role of the epidermal growth factor (ErbB) family of receptors and their ligands in regulating AR activity during prostate cancer progression is currently a focus of intense investigation. This receptor family includes four members: EGFR (ErbB1), ErbB2 (Neu, HER2), ErbB3 (Her3) and ErbB4 (Her4). All EGFR family members contain an extracellular ligand binding domain, a transmembrane region important in regulating receptor activity, and a cytoplasmic tyrosine kinase domain. ErbB3 lacks tyrosine kinase activity due to amino-acid substitutions in the conserved kinase domain (Kirschbaum and Yarden, 2000). ErbB receptors have been implicated in the pathogenesis and progression of many types of human cancers and therapies directed against these receptors are in clinical use (Yarden, 2001). An extensive body of work demonstrating cross talk between ErbB receptors and their ligands and the AR in prostate cancer has evolved (El Sheikh *et al*, 2003). For example, the EGF receptor is overexpressed in both benign prostatic hypertrophy (BPH) and prostate cancer (De Miguel *et al*, 1999). EGFR overexpression is observed in patients during a conversion to androgen independent growth (Olapade-Olaopa *et al*, 2000; Hernes *et al*, 2004). The role of ErbB2 in rendering cells androgen independent or more sensitive to extremely low levels of androgen has been demonstrated both *in vitro* and in animal models (Craft *et al*, 1999; Yeh *et al*, 1999). ErbB2 is also required for IL-6 activation of AR signalling (Qiu *et al*, 1998). However, studies of the role of ErbB2 in clinical prostate cancer remain inconclusive (Grossmann *et al*, 2001), and initial clinical trials indicate that the anti-ErbB2 antibody Herceptin (trastuzumab) does not show significant clinical activity as a single agent (Ziada *et al*, 2004). ErbB3 has not been as extensively studied, but analysis of clinical prostate cancer specimens indicates that overexpression of ErbB3 has been linked to a less favourable prognosis (Leung *et al*, 1997).

EGF-like ligands have been shown *in vitro* and *in vivo* to stimulate growth of prostate cancer cells. For example, *in vitro* AR is activated in a ligand independent manner by EGF (Culig *et al*, 1994). Torring *et al* (2003) recently showed that LNCaP cells constitutively express EGF ligands and that ErbB1 activity is necessary for androgen-induced proliferation. Endogenous stromal derived factors such as Heparin binding-EGF attenuate the response of AR to its ligands, resulting in androgen independent growth of LNCaP cells (Adam *et al*, 2002). By contrast, the ErbB3/4 ligand HRG is highly expressed in basal, luminal and stromal cells of the normal prostate, but not in prostate tumours (Lyne *et al*, 1997). Further, HRG protein is not detected in the prostate cancer cell lines LNCaP, DU-145 or PC-3 (Grasso *et al*, 1997a; Lyne *et al*, 1997). HRG mRNA could be detected in these cell lines only by RT–PCR in one study (Lyne *et al*, 1997), but was not detected in another (Grasso *et al*, 1997a). HRG inhibits growth and induces differentiation of AR positive, ErbB1–3 positive LNCaP cells, but has little effect on proliferation of ErbB1–3 positive, AR negative DU 145 and PC-3 cells (Grasso *et al*, 1997b; Lyne *et al*, 1997). In addition, HRG induces the expression of the tumour suppressor p53 and the CDK inhibitor p21 in LNCaP cells (Bacus *et al*, 1996). Most recently, Tal-Or *et al* (2003) have demonstrated that HRG activates ErbB2/3 heterodimers and induces apoptosis of LNCaP cells. These combined findings suggest that HRG signals may contribute to growth restriction or differentiation of prostate epithelia.

Our laboratory has recently demonstrated that a protein Ebp1, isolated by its binding to HRG's cognate receptor ErbB3 (Yoo *et al*, 2000), binds AR *in vitro* and *in vivo* (Zhang *et al*, 2002). Ebp1 is expressed in both normal prostate epithelial cells and in the prostate cancer cell lines LNCaP, DU-145 and PC-3 (Xia *et al*, 2001b). Ectopic expression of *ebp1* inhibits ligand-mediated transcriptional activation of both artificial and natural AR regulated promoters in COS cells transfected with wild-type AR and in LNCaP cells that express a mutant AR. The transcription of the endogenous PSA gene is also decreased in LNCaP cells stably transfected with Ebp1 (Zhang *et al*, 2002). However, the effect of Ebp1 on transactivation of wild-type AR in prostate cancer cells was not established. The purpose of the present study was to extend our finding that Ebp1 represses AR transactivation and to determine if the ErbB3 ligand, HRG, affects Ebp1's interactions with AR.

## MATERIALS AND METHODS

### Cell culture

All cell lines except PC-3 AR were obtained from the American Type Culture Collection (Manassas, VA, USA). PC-3 AR cells (Long *et al*, 2000) were a gift of Dr Angela Brodie. Cells were maintained at 37°C in a humidified atmosphere of 5% CO_2_ in air. Cell lines were routinely cultured in RPMI 1640 media supplemented with 10% foetal bovine serum (FBS) (Sigma, St Louis, MO, USA).

### Plasmids

The PSA reporter luciferase construct was a gift from Dr Martin Gleave and contains −630/+12 of the 5′ PSA flanking region. The Probasin (−285/+32) luciferase reporter and the pSG5-hAR expression construct were gifts of Dr O Janne. The MMTV-luciferase plasmid was obtained from Dr Joseph Fondell (Wang and Fondell, 2001). The *ebp1* expression construct has been previous described (Xia *et al*, 2001a).

### Immunoprecipitation, GST-pulldowns and Western blot analysis

To measure ErbB3–Ebp1 interactions, LNCaP cells were incubated overnight in serum-free RPMI-1640 media. Where indicated, cells were treated with 20 ng ml^−1^ of HRG *β*1 (R&D Systems, Mpls, MN, USA) for the indicated times. Cell lysates were prepared and immunoprecipitated as described previously (Fernandes *et al*, 1999). Briefly, cells were lysed with buffer containing 50 mM HEPES (pH 7.5), 1 mM EDTA, 150 mM NaCl, 1% Triton X-100 and Complete™ protease inhibitor. Protein concentrations were measured using a detergent compatible kit (BioRad, Hercules, CA, USA). Cell lysates were precleared with Protein A/Protein G agarose and immunoprecipitated for 4 h at 4°C with 2 *μ*g of a monoclonal antibody directed against ErbB3 (Santa Cruz Biotechnology, Santa Cruz, CA, USA) and 20 *μ*l packed Protein A/G agarose beads. The immunoprecipitates were washed and resuspended in Laemmli sample buffer. Proteins were resolved by SDS–PAGE. After electrophoresis, the proteins were transferred onto Immobilin-P membranes, and immunoblotted as described (Xia *et al*, 2001b) using a monoclonal antibody to ErbB3 (Santa Cruz) or a rabbit polyclonal antibody that detects both phosphorylated and unphosphorylated forms of Ebp1 (Xia *et al*, 2001b) (Upstate, Lake Placid, NY, USA). To measure the association of endogenous AR and endogenous Ebp1, LNCaP cells, growing in complete media, were switched to phenol-red free RPMI 1640 containing 1% charcoal stripped calf serum (CSS) (Sigma) and 10^−8^ M R1881 (NEN, Boston, MA, USA) for 24 h. Cells were then stimulated with or without HRG *β*1 (R&D Systems, Mpls, MN, USA) for 1 h. Cell lysates were immunoprecipitated as described above using the polyclonal antibody to Ebp1. Western blot analysis was performed using a monoclonal antibody to AR (Santa Cruz) or the Ebp1 antibody.

### Luciferase reporter assays

Cells (5 × 10^4^) were plated in 12-well plates in complete media. When cells reached 50–60% confluence, they were transfected using the Fugene-6 Reagent (Roche, Indianapolis, IN, USA) according to the manufacturer's instructions. Cells were transfected with 0.5 *μ*g of the indicated reporter plasmids, 0.5 *μ*g of pSG5-hAR (where specified), and 0.5 *μ*g of pcDNA3 or wild-type *ebp1* expression plasmids and 5 ng of the TK-Renilla plasmid (Promega, Madison, WI, USA) as an internal control. Complete medium was replaced 24 h after transfection with phenol red free RPMI 1640 with CSS with or without R1881 (10^−8^ M) (Sadar and Gleave, 2000). Luciferase activity was determined using the Promega Dual luciferase assay kit as described by the manufacturer. The levels of luciferase activity were normalised using the renilla luciferase as an internal control. The ratio of luciferase activity to the renilla control derived from cells that were transfected with vector alone and not treated was given a Relative Luciferase Activity value of 1. All values presented in the individual figures were derived by comparison to this ratio observed in control cells. Transfection efficiency was approximately 30% as judged by parallel experiments using the EGFP-N1 plasmid (Clontech, Palo Alto, CA, USA). All transfection experiments were carried out in triplicate wells.

### Gene silencing with small interfering RNAs

The siRNA oligonucleotides were purchased from Dharmacon Research Inc (Lafayette, CO, USA). COS-7 cells were cultured in 12-well plates until 60% confluent. Cells in 1 ml of antibiotic-free culture media were transfected with 60 nM final concentration of annealed oligonucledotides using Lipfectamine 2000 according to the manufacturer's instructions. The Ebp1 siRNA sequences corresponded to the coding regions beginning at nucleotides 476 and 995 (Genbank accession number U87954). The targets sequences were AAGCGACCAGGAUUAUAUUCU and AAGUGAGGUGGAAAGGCGUUU respectively. These sequences do not match any other human genomic sequences as determined by BLAST analysis using the NCBI Website. Scrambled oligonucleotides of these sequences were used as negative controls. The next day, cells were transfected with an expression construct for wild-type AR and the MMTV-luciferase and TK plasmids using Fugene-6.

### Statistical analysis

Results were analysed using a two-tailed Students*t*-test. Significance was established at *P*⩽0.05.

## RESULTS

### Ebp1 inhibits transactivation of wild-type AR in prostate adenocarcinoma cell lines

**Figure 1:**
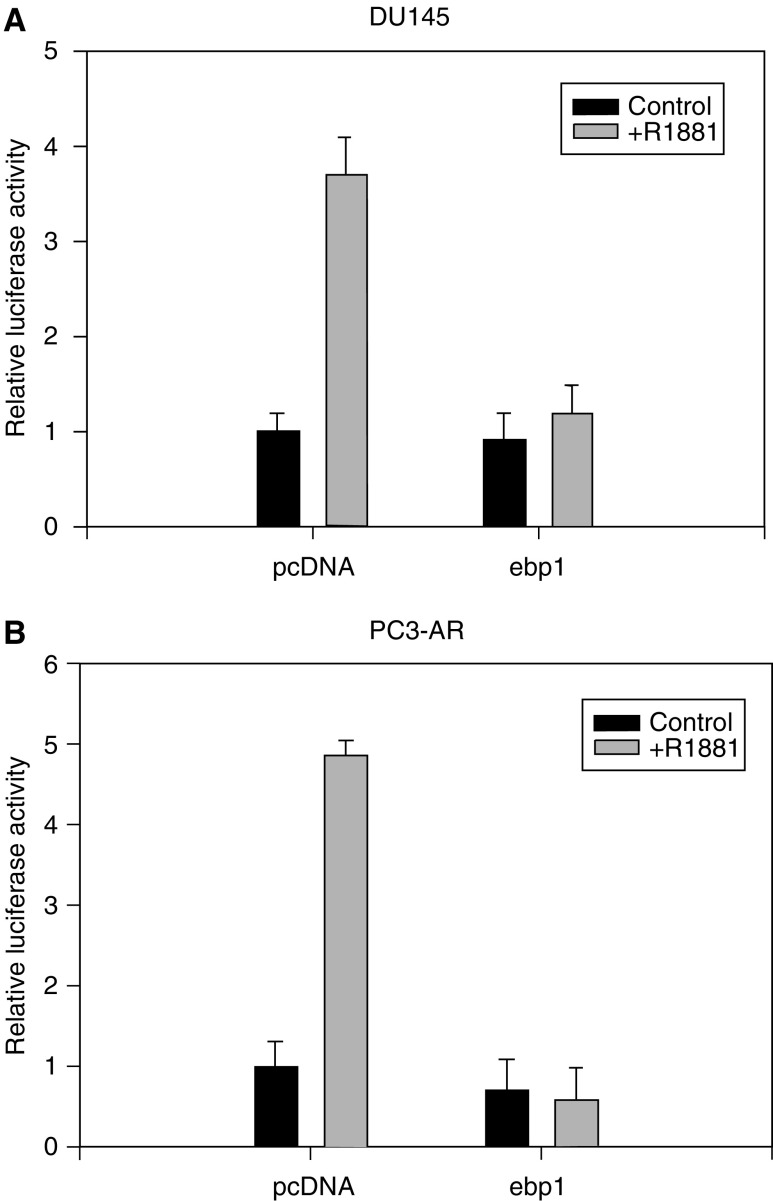
Ebp1 inhibits the transcriptional activity of the MMTV promoter in prostate cancer cell lines expressing wild-type AR DU145 (**A**) or PC3-AR cells (**B**) were transfected with an MMTV-luciferase reporter plasmid and pcDNA or pcDNA-*ebp1* expression plasmids where indicated. DU145 cells were also transfected with a wild-type AR expression plasmid. At 24 h after transfection, cells were switched to phenol-red free RPMI 1640 media with 1% CSS containing R1881 (10^−8^ M) or vehicle control. After 16 h, luciferase activity was measured. Each point represents mean±s.e. of triplicate wells. Representative of three experiments.

We previously demonstrated that Ebp1 inhibited transactivation of the artificial ARE_2_DS promoter in COS cells transfected with wild-type AR, and the PSA promoter in LNCaP cells that harbor a mutant AR (Zhang *et al*, 2002). We were interested in determining if Ebp1 represses wild-type AR in prostate cancer cell lines. We therefore tested the ability of Ebp1 to repress AR-mediated transcription of the MMTV-luciferase reporter plasmid in androgen-independent DU145 cells transiently transfected with a wild-type AR and PC-3 AR cells stably transfected with wild-type AR (Long *et al*, 2000). Cells were transfected with the MMTV luciferase reporter construct, *ebp1* or the pcDNA vector control, and in the case of DU145 cells, the expression construct for AR. After 24 h, the cells were stimulated with either R1881 or vehicle for 16 h. Cells were harvested and monitored for dual luciferase activity. Transfection of *ebp1* at 0.5 *μ*g per individual well routinely results in a two- to three-fold increase in Ebp1 expression levels as determined by Western blot analysis (data not shown). As expected, R1881 stimulated luciferase activity four- to five-fold in DU145 and PC-3 AR cells (Figure 1). Ectopic expression of *ebp1* reduced AR transactivation to basal levels in both cell types.

**Figure 2:**
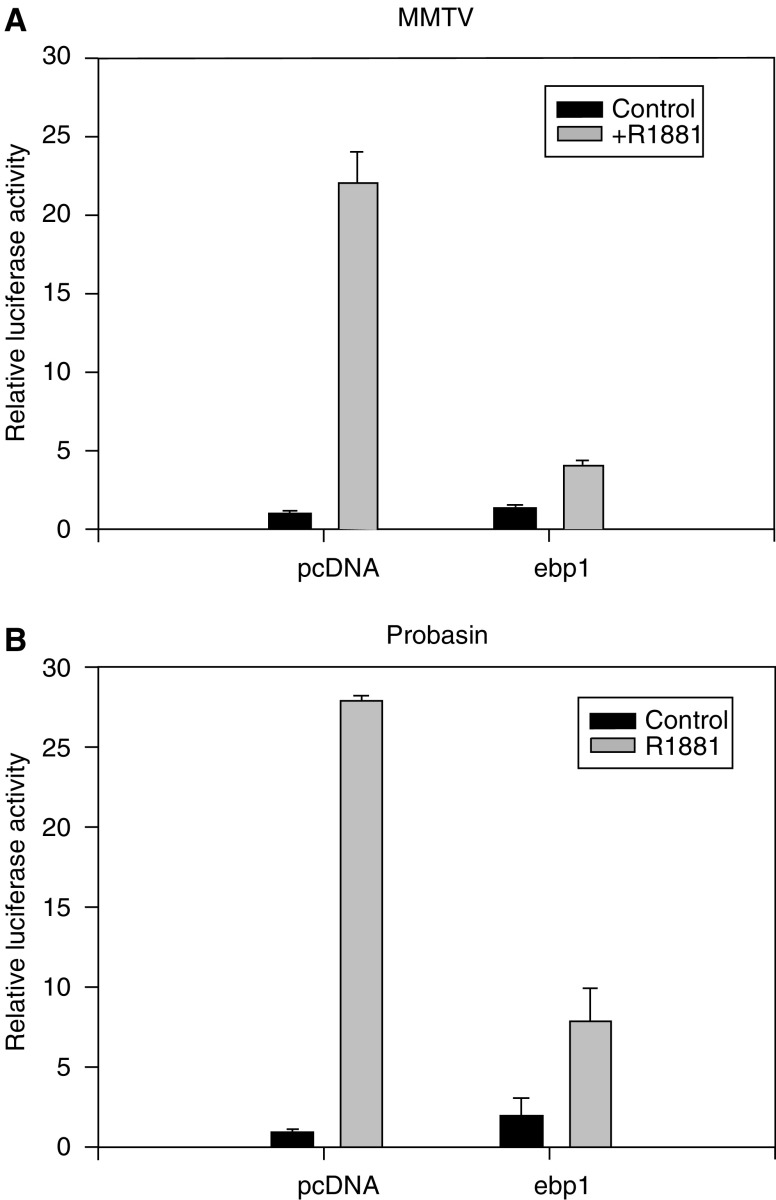
Ebp1 inhibits transcriptional activity of native AR target promoters in LNCaP cells. LNCaP cells were transfected with the MMTV (**A**) or probasin reporter constructs (**B**) and 0.5 *μ*g of pcDNA3 or the wild-type *ebp1* expression plasmid. At 24 h after transfection, cells were treated with R1881 as described in Figure 1 for an additional 16 h and relative luciferase activity was measured. Each point represents mean±s.e. of triplicate wells. Representative of two experiments.

Corepressors may have dissimilar effects on the activity of natural AR regulated promoters due to differential binding of the AR to androgen response elements within those promoters (Claessens *et al*, 2001). We had previously demonstrated that Ebp1 represses both exogenous and endogenous PSA promoter activity in LNCaP cells (Zhang *et al*, 2002). We therefore tested the ability of Ebp1 to repress the MMTV-luc and probasin native androgen responsive promoters. LNCaP cells were stimulated with R1881 and the induction of luciferase activity in the presence and absence of exogenous *ebp1* was measured. Probasin and MMTV promoters were strongly activated by R1881. AR activation of both promoters was reduced significantly (*P*⩽0.05) with the ectopic expression of *ebp1* (Figure 2). However, a small but significant increase in AR activity after R1881 treatment was noted even in the presence of *ebp1*.

**Figure 3:**
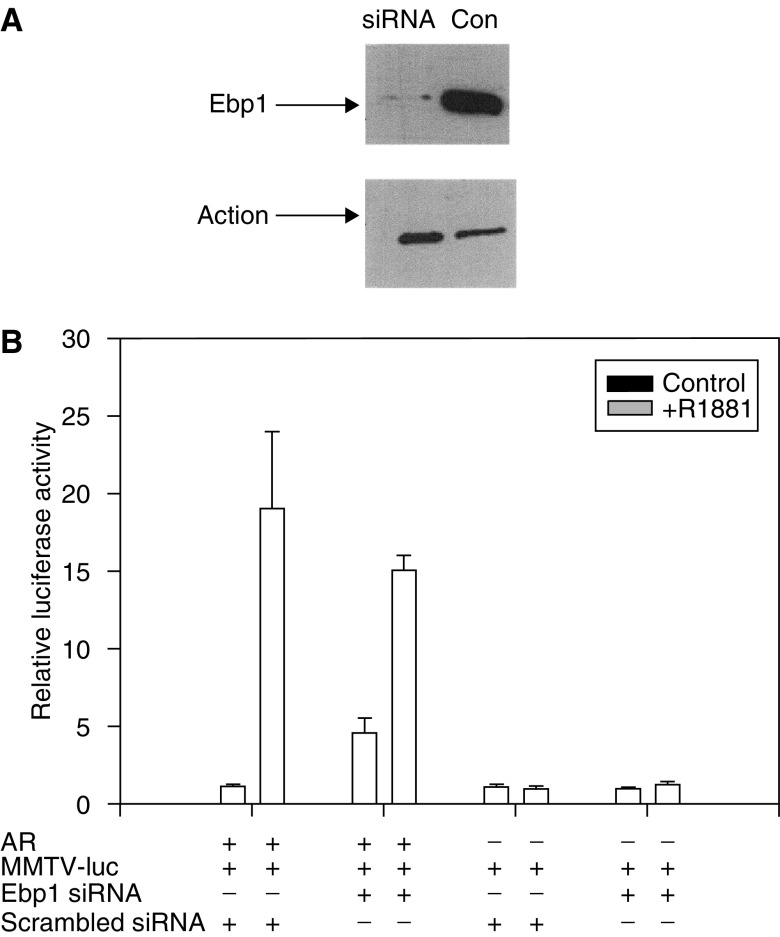
The effect of Ebp1 specific small interfering RNA on transactivation of AR in COS-7 cells. COS-7 cells were transfected with scrambled siRNAs (Con) or siRNAs (siRNA) directed towards *ebp1* cDNA sequences 476 and 995 using Lipofectamine 2000. The next day the cells were transfected with the wild-type AR expression plasmid, MMTV-luc and RL-TK. After 24 h, the cells were switched to phenol-red free RPMI 1640 media with 5% CSS with or without R1881. Cell lysates were harvested for Western blot analysis and luciferase activity 16 h later. (**A**) The expression of Ebp1 was analysed by Western blotting. Cell lysates were resolved by SDS–PAGE, proteins transferred onto PVDF membranes and filters blotted with Ebp1 or actin antibodies as indicated. (**B**) Effect of Ebp1 siRNA on AR promoter activity. Aliquots of the cells harvested in (**A**) were assayed for luciferase activity. Each point represents mean±s.e. of triplicate wells. Representative of three experiments.

We next wished to determine if endogenous Ebp1 was important in AR signalling. COS-7 cells were first transfected with siRNA targeted to two regions in the Ebp1 cDNA as described in the Materials and Methods. Cells were then transfected with the AR expression plasmid, and the MMTV-luciferase reporter construct the next day. Cells were stimulated with R1881 on day 3 and lysates collected on day 4. The results of Western blotting experiments showed that transfection of siRNA directed against Ebp1 reduced proteins levels about 80% at Day 4 (Figure 3A). This decrease was not observed in cells transfected with scrambled oligos. Decreased expression of Ebp1 resulted in a significant (*P*⩽0.05) 3.5-fold increase in the luciferase activity of the MMTV promoter in the absence of androgen. No such stimulation was observed in cells lacking AR. R1881 stimulation of the reporter plasmid was decreased by inhibition of Ebp1 expression, but this change was not significant at the *P*⩽0.05 level (Figure 3B). These results suggest that Ebp1 may be important in repression of AR in the absence of androgen.

### HRG regulates the binding of Ebp1 to ErbB3 and AR

**Figure 4:**
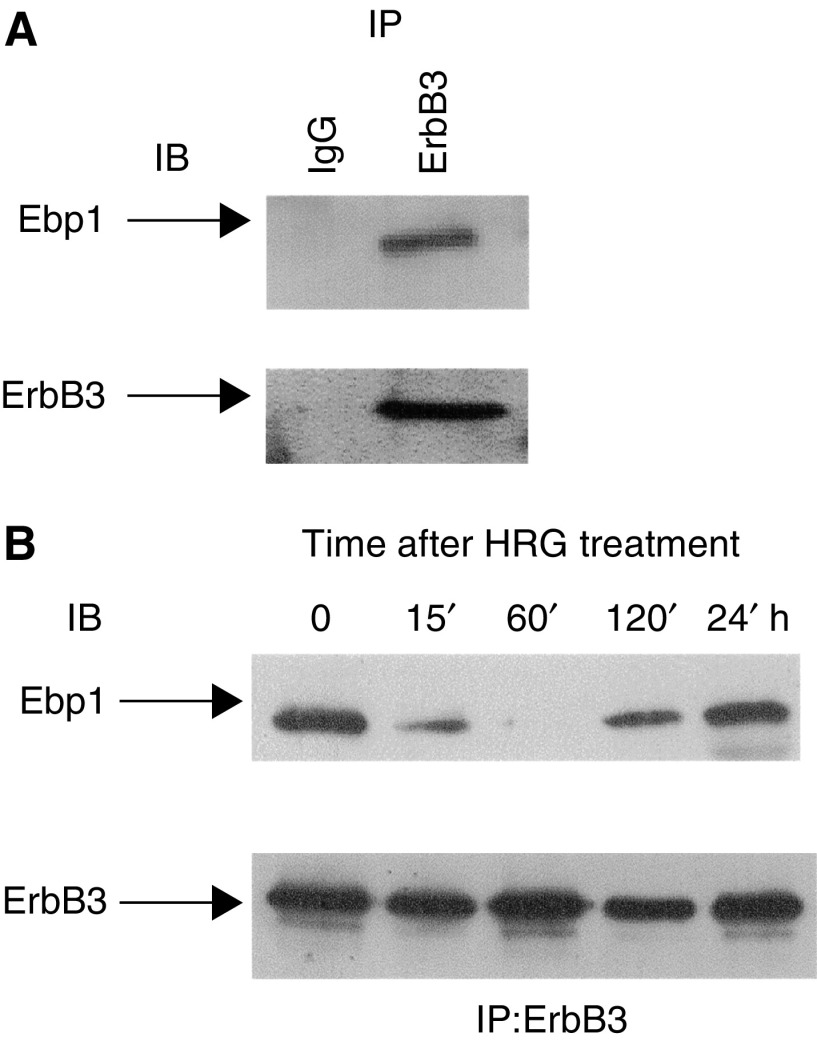
*In vivo* interaction of Ebp1 with ErbB3 (**A**) Ebp1 associates with ErbB3 in LNCaP cells. LNCaP cell lysates were immunoprecipitated with mouse isotype control IgG (lane 1) or an anti-ErbB-3 monoclonal antibody (lane 2) and analysed by sequential immunoblotting (IB) with either Ebp1 (top panel) or ErbB3 (bottom panel) antibodies. (**B**) HRG-induced dissociation of Ebp1. LNCaP cells were left untreated (0) or stimulated with HRG (20 ng ml^−1^) for the times indicated. ErbB-3 associated Ebp1 was analysed by ErbB-3 immunoprecipitation (IP) and sequential immunoblotting (IB) with antibodies against Ebp1 (top) and ErbB-3 (bottom).

We next determined if ErbB3 could bind Ebp1 in human prostate cell lines as it does in breast carcinoma cells (Yoo *et al*, 2000) and if HRG could affect this binding. Lysates of serum starved LNCaP cells were incubated with either a mouse monoclonal antibody to ErbB3 or control IgG. Proteins were resolved by SDS–PAGE and immunoblotted with antibody to Ebp1. Ebp1 was found in ErbB3, but not control, immunoprecipitates (Figure 4A). Next, we determined if the binding of Ebp1 to ErbB3 could be regulated by HRG. LNCaP cells were serum starved and treated with HRG (20 ng ml^−1^) for 0, 15, 60 and 120 min and 24 h. Cell lysates were immunoprecipitated with antibody to ErbB3 as described. Ebp1 was found in ErbB3 immunoprecipitates of untreated cells (Figure 4B). There was a decrease in the level of Ebp1 associated with ErbB3 starting at 15 min. No Ebp1 was found in the Erb3 immunoprecipitates 60 min after treatment. Binding was increased 2 h after treatment and by 24 h after HRG treatment, Ebp1 binding to ErbB3 was restored.

**Figure 5:**
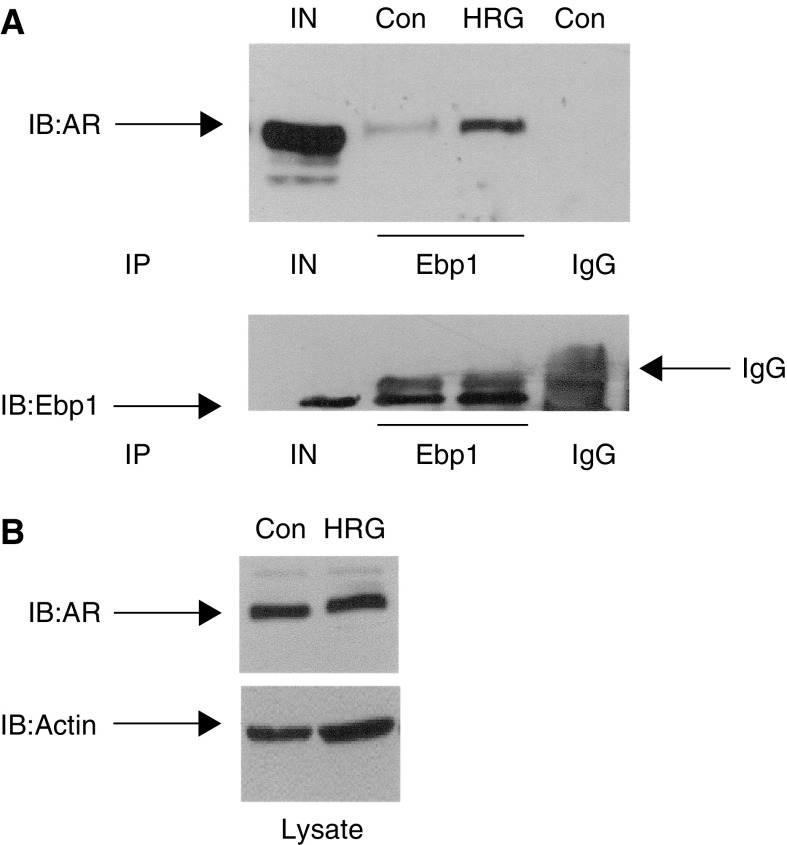
HRG increases the binding of AR to Ebp1 in LNCaP cells. (**A**) LNCaP cells growing in complete media were switched to phenol red free RMPI 1640 containing 1% CSS and 10^−8^ M R1881 overnight. Cells were then incubated in the absence (Con) or presence of HRG *β*1 (HRG) (20 ng ml^−1^) for 1 h. Cell lysates were then immunoprecipitated (IP) with antibody to Ebp1 (Ebp1, lanes 2 and 3) or preimmune IgG (IgG) (lane 4). Immunoblots (IB) were analysed using a monoclonal antibody to AR (upper panel) or a polyclonal antibody to Ebp1 (lower panel) as indicated. IN=input (5% of total) used for immunoprecipitation (lane 1). IgG indicates the heavy chain of IgG detected in Ebp1 immunoblots of the Ebp1 immunoprecipitates. (**B**) Effects of HRG on AR protein levels. Control (Con) or HRG treated (HRG) LNCaP cells were prepared as described in (**A**). Total cell lysates were resolved by SDS–PAGE, proteins transferred onto PVDF membranes, and Western blot analysis performed using monoclonal antibodies to AR or actin as indicated.

As HRG treatment resulted in the release of Ebp1 from ErbB3, we next determined whether HRG could regulate the association of endogenous Ebp1 with endogenous AR. LNCaP cells, growing in complete media, were switched into phenol-red free RPMI 1640 media with 1% CSS and R1881 (10^−8^ M) overnight. Cells were then treated with 20 ng ml^−1^ of HRG *β*1 for 1 h, a time when we could not detect Ebp1 in ErbB3 immunoprecipitates. Cell lysates were immunoprecipitated with the Ebp1 antibody. Western blot analysis of the immunoprecipitates indicated that HRG treatment enhanced the interaction of Ebp1 with AR (Figure 5A). Examination of cell lysates revealed that HRG treatment did not increase the level of AR protein at this 1 h time point (Figure 5B).

**Figure 6:**
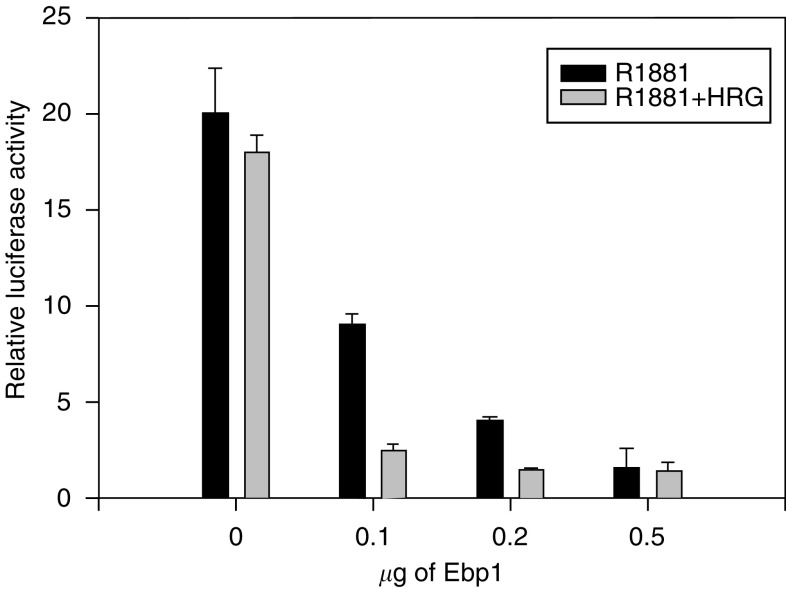
HRG enhances Ebp1 mediated repression of the AR promoter LNCaP cells were transfected with the MMTV-luciferase reporter plasmid (0.5 *μ*g) and an *ebp1* expression plasmid or a pcDNA vector control at the indicated amounts. After 24 h, cells were switched to RPMI 1640 phenol-red free media with 5% CSS and R1881 with or without HRG *β*1 (20 ng ml^−1^). Cell lysates were assayed for luciferase activity 16 h later. The relative luciferase activity in the absence of R1881 was set at 1 for cells that received the vector control. All activities presented in the graph were derived by comparison to that value. Neither HRG nor *ebp1* affected luciferase activity under these basal conditions. Each point represents mean±s.e. of triplicate wells. Representative of two experiments.

HRG enhances Ebp1 transcriptional repression. We reasoned that if HRG could change the association of Ebp1 with AR, it could affect Ebp1 induced repression of AR transactivation. LNCaP cells were transiently transfected with the MMTV luciferase reporter plasmid and limiting amounts of an *ebp1* expression construct. *Ebp1* at low concentrations (0.1 *μ*g) reduced AR luciferase activity 55%. Maximal inhibition of 90% was observed at 0.5 *μ*g of the *ebp1* plasmid. This was more than the 80% inhibition previously observed (Figure 1) and probably due to changes in transfection efficiencies as different batches of cells and plasmids were used in these different experiments. Concentrations of HRG (20 ng ml^−1^), previously demonstrated to increase association of Ebp1 and AR, significantly (*P*⩽0.05) enhanced Ebp1-mediated repression at low (0.1 and 0.2 *μ*g) amounts of the Ebp1 plasmid (Figure 6).

## DISCUSSION

We have previously established that Ebp1, a protein cloned in our laboratory via its interactions with the ErbB3 receptor, inhibits AR-mediated transcription and growth of the AR positive LNCaP cell line (Zhang *et al*, 2002). In this report, we confirm and extend our findings by demonstrating that Ebp1 is capable of inhibiting receptor transactivation independent of cell type or AR target promoter. In addition, we demonstrate that HRG, the ErbB3 ligand, stimulated the association of Ebp1 with AR and increased Ebp1 mediated repression of AR activity, providing further evidence for a link between ErbB ligands and AR function.

This study first demonstrated that Ebp1 inhibition of AR transactivation was neither promoter nor cell type specific. We had previously demonstrated that Ebp1 inhibits AR transactivation of the artificial ARE_2_ luciferase reporter in COS cells and the PSA luciferase reporter in LNCaP cells (Zhang *et al*, 2002). However, recent studies have demonstrated that AR mediated gene transcription is influenced by the cell type examined (Kotaja *et al*, 2000; Claessens *et al*, 2001; Holter *et al*, 2002). For example, Holter *et al* (2002) have shown that DAX1 inhibition of PSA and the ARE_2_ reporter was more potent in COS-7 than HeLa cells. This variability has been attributed to the complement of transcription factors and coregulators in different cell types. Therefore, we examined AR transactivation of MMTV-luc in two androgen-independent prostate cancer cell lines, PC3 and DU145, that had been transfected with wild-type AR. Ebp1 inhibited AR-regulated transcription in these androgen-independent cells. Similarly, Cyclin D1 inhibits AR transactivation across a wide variety of both prostate and nonprostate derived cell lines (Petre-Draviam *et al*, 2003). In addition, a number of AR coregulators demonstrate promoter specificity. For example, ARIP3 enhances transcription from minimal AREs, yet represses the probasin promoter (Kotaja *et al*, 2000). Activation of both the PSA and probasin promoters requires interactions of the N and C terminal domains of AR, but this association is not required for activation of MMTV (He *et al*, 2000). Thus, it was important to examine the effects of Ebp1 on different native promoters. Here we show that Ebp1 also inhibited the probasin and MMTV reporters in LNCaP cells as well as PSA as previously demonstrated (Zhang *et al*, 2002). Our previous studies had also shown that inhibition of AR transactivation is specific, as Ebp1 did not affect the estrogen induced responsiveness of an ERE luciferase reporter or the thyroid hormone mediated activity of a TRE luciferase reporter plasmid (Zhang *et al*, 2002). It is of interest to note that while *ebp1* overexpression completely inhibited AR activity in DU145 and PC-3 AR transfected cells, *ebp1* was unable to completely suppress the response to R1881 in LNCaP cells. This discrepancy may have been due to different expression levels of AR in the DU145 and PC-3 cells lines as compared to LNCaP cells, the fact the AR receptor is mutated in LNCaP cells, or to different transfection efficiencies of the *ebp1* plasmid among the three cell lines.

Studies using siRNA demonstrated that inhibition of endogenous Ebp1 expression resulted in increased activity of an androgen regulated reporter construct in the absence of androgens. No such effect was observed in cells not expressing the AR. Thus, the increase in promoter activity in the absence of androgens was mediated via the AR. This finding suggests that Ebp1 may play a role in inhibition of AR signalling in the presence of no or extremely low levels of androgens. Ebp1 has the ability to bind DNA (Zhang and Hamburger, 2004) and histone deacetylases (Zhang *et al*, 2003), and we postulate that Ebp1 might reside on AR regulated promoters in the absence of androgens to inhibit transcription. ChIP assays to demonstrate Ebp1 occupancy on AR promoters are underway in the laboratory. The fact that abolition of Ebp1 protein enhanced basal, but not AR stimulated transcription, is somewhat puzzling in light of the fact that overexpression of Ebp1 inhibits R1881 stimulated, but not basal, transcription of AR regulated genes. It is possible that basal transcription by AR is so low in unstimulated LNCaP cells that Ebp1-mediated repression in the absence of androgens may have gone undetected. Conversely, overexpression of Ebp1 might drive Ebp1 to AR promoters in the presence of androgens. Ebp1 might then recruit HDACS, important in AR transcriptional repression (List *et al*, 1999), to the promoter to inhibit gene transcription. The fact that inhibition of expression of Ebp1 leads to increased transcriptional activation of AR suggests that endogenous Ebp1 may function to regulate AR signalling in prostate cancer cells.

Although Ebp1 was shown to be associated with ErbB3 in breast cancer cells (Yoo *et al*, 2000), the interaction of Ebp1 with ErbB3 in prostate cells had not yet been demonstrated. Here, we determined that Ebp1 could also bind ErbB3 in LNCaP cells. To the best of our knowledge, this is the first demonstration of a direct interaction between ErbB3 and the AR. It is of interest that Bonaccorsi *et al* (2004) recently reported the physical association and subcellular colocalisation of the EGFR with AR in PC3 cells transfected with the AR. These studies further support the concept that ErbB receptors and AR interact *in vivo*.

We then determined the effects of HRG on Ebp1 function. HRG treatment resulted in dissociation of Ebp1 from the ErbB3 receptor in LNCaP cells. The present studies also demonstrate that HRG regulated the interaction of Ebp1 with the AR receptor. First, we demonstrated that endogenous Ebp1 associated with endogenous AR *in vivo*. However, the efficiency of the Ebp1 : AR interaction was relatively low in the absence of HRG. HRG treatment of LNCaP cells for 1 h enhanced the association of Ebp1 with AR in LNCaP cells. The basis of the increased association of Ebp1 with AR after HRG treatment is not known. We have found that HRG increases phosphorylation of Ebp1 in breast cancer cells (Lessor *et al*, 2000) and studies are underway in the laboratory to examine if enhanced phosphorylation of Ebp1 increases its binding to AR. The intracellular compartment in which AR and Ebp1 interact in either the presence or absence of HRG is not clear at this time. Immunofluorescence analysis in our hands indicates that both Ebp1 and AR are located in the nucleus and the cytoplasm of LNCaP cells in the absence of HRG and/or R1881 (data not shown).

The fact that HRG enhances the binding of Ebp1 to AR suggests that in the absence of HRG, Ebp1 may not optimally affect AR function. Indeed, HRG potentiated the ability of limiting amounts of Ebp1 to inhibit AR promoter activity. HRG has been previously shown to inhibit growth of AR+ but not AR− prostate cancer cell lines (Grasso *et al*, 1997b; Lyne *et al*, 1997). Similarly, Abreu-Martin *et al* (1999) found that MEKK-1, a downstream mediator of HRG signalling, induced apoptosis of AR+, but not AR−, prostate cancer cell lines. We hypothesise that Ebp1 may be one mediator of the effect of HRG on AR function. In the presence of low concentrations of HRG, such as has been observed in prostate cancer tissues (Lyne *et al*, 1997), the activity of Ebp1 may be suboptimal, resulting in increased AR signalling. Thus, although Ebp1 may be present in prostate cancer cells (Xia *et al*, 2001b), it may not be fully functional.

In summary, Ebp1 is a potent corepressor of AR with broad specificity. Ebp1 maintains its corepressor activity independent of cell type and promoter examined. Thus, Ebp1 joins a small but growing group of AR corepressors (Culig *et al*, 2003). The fact that interactions of Ebp1 and AR were regulated by HRG suggests a link between the HRG-generated growth inhibitory signals transduced through the ErbB3 receptor and the AR receptor. Further studies are needed to characterise the interactions of ErbB3, Ebp1 and AR in the progression of prostate cancer.
